# The predictor role of metacognition and emotion recognition in internet gaming disorder among late adolescents

**DOI:** 10.1192/j.eurpsy.2021.2169

**Published:** 2021-08-13

**Authors:** O. Aydın, P. Ünal-Aydın, Y. Arslan, M. Güçlü, S. Çakiroğlu

**Affiliations:** 1 Psychology, International University of Sarajevo, Faculty of Arts and Social Sciences, Sarajevo, Bosnia and Herzegovina; 2 Child And Adolescent Psychiatry, İstanbul Medeniyet University Göztepe Education and Research Hospital, Istanbul, Turkey

**Keywords:** internet gaming, metacognition, emotion recognition, adolescence

## Abstract

**Introduction:**

Internet gaming is acknowledged as a common leisure activity among adolescents yet only a little known about the pscyhodevelopmental roots. Emotion recognition and metacognition which are proved to be determinants in behavioral disorders may be considered salient factors in also internet gaming disorder (IGD).

**Objectives:**

The research to date has focused on psychological comorbidities rather than risk factors (e.g. dysfunctional metacognitive beliefs, emotion recognition deficits), whereas, improved early intervention would be more likely if risk factors were well defined, especially before the onset of the illness. The objective of this study was to investigate these areas by analyzing associations between metacognitive beliefs, emotion recognition, and IGD among late adolescents with tendency for pathological gaming behavior.

**Methods:**

806 high school students were recruited and instructed to take Internet Gaming Disorder Scale (IGDT), Meta-Cognitions Questionnaire for Children and Adolescents (MCQ-C) and Reading the Mind in the Eyes Test - Children’s Version (RMET).

**Results:**

Mean comparisons corresponding to IGD risk potential showed that positive meta-worry and superstitious, punishment, and responsibility beliefs of MCQ-C were significantly higher in the risky group, whereas; RMET neutral subtest was significantly higher in the non-risky group. Additionally, a positive correlation was found in all subtests of MCQ-C, RMET positive emotions and IGD. Regression analysis revealed that RMET positive subtest and positive meta-worry of MCQ-C predict IGD risk.

**Conclusions:**

The findings of the study partially corroborated the early results found among early adolescents; however, they also indicated the requirement of distinct therapeutic approach for cognitive interventions of IGD in late adolescence period.

**Disclosure:**

No significant relationships.

